# Comparison of the Accuracy of Modified CT Severity Index Score and Neutrophil-to-Lymphocyte Ratio in Assessing the Severity of Acute Pancreatitis

**DOI:** 10.7759/cureus.17020

**Published:** 2021-08-09

**Authors:** Hasan Tahir, Sheeraz Rahman, Zahid Habib, Yusra Khan, Saleha Shehzad

**Affiliations:** 1 Department of Plastic and Reconstructive Surgery, Liaquat National Hospital and Medical College, Karachi, PAK; 2 Department of General Surgery, Liaquat National Hospital and Medical College, Karachi, PAK; 3 General Surgery, Karachi Medical and Dental College, Karachi, PAK; 4 Department of Radiology, Liaquat National Hospital and Medical College, Karachi, PAK

**Keywords:** acute pancreatitis, modified ct severity index score (mctsi), revised atlanta 2012, severity, neutrophil-to-lymphocyte ratio (nlr)

## Abstract

Background

Acute pancreatitis is an acute gastrointestinal emergency with significant morbidity and mortality if not treated. It can lead to local as well as systemic complications and has a prevalence of 51.07%. Laboratory investigations such as amylase and lipase and ultrasound scan are typically used for the diagnosis. A contrast-enhanced CT scan is considered the gold standard. Both laboratory and radiological investigation-based scoring systems have been reported in the literature and are in practice. However, these modalities demand several laboratory investigations and are expensive. Our study aims to determine the congruency of the neutrophil-to-lymphocyte ratio (NLR) and the modified CT severity index score (MCTSI) with the revised Atlanta classification in assessing the severity of acute pancreatitis. In addition, the accuracy of NLR and MCTSI is determined. The secondary objective is to determine whether NLR can predict the severity of acute pancreatitis to the same extent as MCTSI through expensive radiological imaging and other clinical scoring systems through a list of investigations.

Methodology

The data for this study were collected retrospectively and patients with a diagnosis of acute pancreatitis were included through the nonprobability convenience sampling method. All patients underwent relevant laboratory workup (including complete blood count) and radiological workup (including CT scan) during their hospital stay. The main outcome measures were sensitivity, specificity, and accuracy of NLR and MCTSI, and the congruency of these with the revised Atlanta classification in assessing the severity of acute pancreatitis.

Results

A total of 166 patients with acute pancreatitis were included, of which 107 (64.45%) were males and 59 (35.55%) were females, with a mean age of 43.7. The sensitivity, specificity, and accuracy of NLR were 67%, 90.9%, and 76%, respectively, whereas the sensitivity, specificity, and accuracy of MCTSI were 95%, 13.6%, and 62%, respectively. The area under the curve for NLR was 0.855 whereas that for MCTSI was determined to be 0.645.

Conclusions

NLR has a good concordance with the revised Atlanta classification and assesses the disease severity, especially in moderate-to-severe cases of acute pancreatitis compared to MCTSI. In addition, NLR can be used in acute and/or resource-poor settings to predict the severity of acute pancreatitis.

## Introduction

Acute pancreatitis is an acute gastrointestinal emergency that predominantly presents with rapid onset of severe abdominal pain, associated with nausea and vomiting. Alcohol abuse and gallstones are among the most common causes. Acute pancreatitis can be diagnosed clinically, biochemically by raised serum amylase and lipase levels, with lipase being more specific, and radiologically by imaging. It can lead to local as well as systemic complications. According to Rehan et al., the prevalence of acute pancreatitis is 51.07%. According to Bhanou et al. [1], Banks et al. [2], and Büchler et al. [3], early recognition of at-risk patients is crucial as mortality of severe acute pancreatitis reaches up to 20%. In 1992, the Atlanta classification was proposed in which pancreatitis was categorized into mild and severe. The classification was further revised in 2012 and categorized acute pancreatitis into mild, moderate, and severe based on a modified marshall scoring system [4,5]. In addition, the revised Atlanta classification classified acute pancreatitis based on the presence or absence of local complications and the presence or absence of organ failure [5].

Several scoring systems and predictors of morbidity and mortality are routinely utilized, including the Ranson score, the Marshall score, the Acute Physiologic Assessment and Chronic Health Evaluation II (APACHE II) score, the Bedside Index for Severity in Acute Pancreatitis (BISAP) score, the sequential organ failure assessment (SOFA), and the Glasgow Coma Scale score [6]. These scoring systems are complex and largely dependent on laboratory investigations which increase resource utilization. Additionally, a few of these scoring systems require reassessment.

Recently, the neutrophil-to-lymphocyte ratio (NLR) has been recognized as a predictor of disease severity and outcome in acute pancreatitis and various other diseases [7]. Alteration in this ratio reflects the severity of the disease, that is, an increase in the neutrophil count indicates acute inflammatory response and a decrease in the lymphocyte count reflects deterioration in overall health [8]. NLR has a sensitivity of 78.12% and a specificity of 70.27% in predicting the severity of acute pancreatitis. An NLR of 1-3 is considered normal, 6-9 mild, 9-18 moderate, and >18 as severe stress.

Imaging techniques such as ultrasound and CT scans can also help in the diagnosis and the determination of the severity of pancreatitis. A contrast-enhanced CT scan is considered to be the gold standard in diagnosing acute pancreatitis. It is sensitive enough to detect pancreatic necrosis and extrapancreatic complications [9]. Among radiological scoring systems, CT severity and modified CT severity index score (MCTSI) are widely adopted in clinical and research settings. MCTSI classifies acute pancreatitis based on radiological findings, that is, the presence or absence of pancreatic inflammation/necrosis and extrapancreatic complications. The sensitivity, specificity, positive predictive value (PPV), and accuracy of MCTSI for detecting moderate/severe disease have been reported to be 100%, 92.3%, 94.4%, and 96.7%, respectively [9].

Various studies have been published considering the different scoring and prognostic systems to assess the severity of acute pancreatitis and prognosis. This study aims to determine the accuracy and congruency between MCTSI and NLR in determining the prognosis of acute pancreatitis considering the revised Atlanta classification as the gold standard.

This study is the first of its kind comparing the three different scoring systems as a predictor of the severity of acute pancreatitis designed to determine whether NLR can be used in the acute setting to predict disease severity when modern imaging techniques are not available and to determine how accurately the two prognostic criteria can predict disease severity considering the revised Atlanta classification as the gold standard. This study aims to determine whether NLR can predict the severity of acute pancreatitis to the same extent as MCTSI and other complex scoring systems, which require extensive investigations and resources often not available in primary healthcare centers and resource-poor areas.

## Materials and methods

This retrospective study was conducted at the Department of General Surgery, Liaquat National Hospital and Medical College in Karachi. After receiving approval from the institutional ethical and research committee, data collection was commenced. Patients admitted with a diagnosis of acute pancreatitis, of either gender, aged 18-75 years, and who underwent contrast-enhanced CT scan at least 72 hours after admission or onset of symptoms for severity assessment during the period January 1, 2016 to December 31, 2019 were included in this study. Medical record of these patients was retrieved using the institutional health management information system, and radiological and laboratory data were retrieved from the respective departments.

Data collected included demographic data, complete blood count information, MCTSI score on contrast-enhanced CT scan, and other related laboratory and radiological data showing the presence or absence of local complications associated with acute pancreatitis and the presence or absence of transient/persistent organ failure. The main outcome measures were diagnostic accuracy of NLR and MCTSI and congruency of NLR and MCTSI with the revised Atlanta classification. Considering a sensitivity of 78.12%, specificity of 70.27%, prevalence of 51.07%, d of 10%, and 95% confidence level, the sample size was calculated to be 166 [1]. All patients who met the inclusion criteria were included in the study via the nonprobability convenience sampling method.

Statistical analysis

Data retrieved were compiled and analyzed using the Statistical Package for Social Sciences version 25.0 (IBM Corp., Armonk, NY). Descriptive statistics, congruency of the three scoring systems, accuracy, and receiver operating characteristic (ROC) curve were computed to determine the sensitivity and specificity. In this study, the prevalence of acute pancreatitis was considered to be 51.07%.

## Results

Of the total 166 patients included in the study, 107 (64.45%) were males and 59 (35.55%) were females. The mean age of the patients was 43.7 ± 16.67. A total of 45 patients had known comorbidities such as diabetes and hypertension. The most common symptom was abdominal pain, followed by nausea and vomiting in many patients. All patients had raised amylase and lipase levels, which are specific and sensitive markers for acute pancreatitis. The overall mean NLR for the study population was 11.72 ± 9.52 (minimum: 1; maximum: 47). The mean NLR for only mild cases was 5.88 ± 2.115 while for moderate and severe cases together was 19.15 ± 10.102. No remarkable difference was seen between NLR for males (11.41 ± 9.505) versus females (11.89 ± 9.571). The mean NLR for mild, moderate, and severe cases for females was 5.74, 12.86, and 35.33, respectively. Similarly, the mean NLR for mild, moderate, and severe cases for males was 5.95, 13.17, and 27.27, respectively. This finding points toward the observation that high NLR was observed for severe cases among females compared to severe cases among males, which needs to be further investigated to determine the probable cause of high NLR among females. On comparing NLR with the revised Atlanta classification for acute pancreatitis, we found that it is an excellent predictor of severity for mild cases, a good predictor for severe cases, and a fair predictor for moderate cases. Similarly, when the MCTSI was compared with the revised Atlanta classification for acute pancreatitis, we found that it is a poor predictor of severity for mild cases, a good predictor for moderate cases, and an excellent predictor for severe cases (Table 1).

**Table 1:** Degree of congruency among the three scores for acute pancreatitis, namely, the revised Atlanta classification, NLR, and MCTSI score. NLR: neutrophil-to-lymphocyte ratio; MCTSI: modified CT severity index score

Revised Atlanta classification					
Mild = 66		Moderate = 77		Severe = 23	
NLR	MCTSI	NLR	MCTSI	NLR	MCTSI
90.9%	13.6%	45.5%	46.8%	60.9%	82.6%

**Figure 1:**
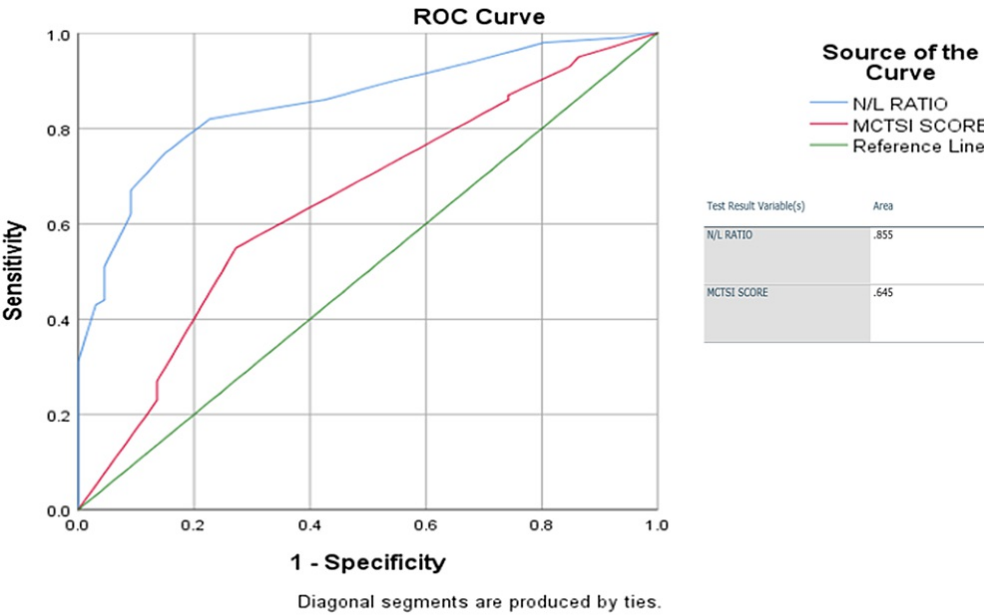
ROC curve demonstrating the comparison of the sensitivity of NLR versus MCTSI. ROC: receiver operating characteristic; NLR: neutrophil-to-lymphocyte ratio; MCTSI: modified CT severity index score

In our study, the sensitivity, specificity, and accuracy of NLR were 67%, 90.9%, and 76%, respectively, whereas the sensitivity, specificity, and accuracy of MCTSI were 95%, 13.6%, and 62%, respectively. On plotting the ROC curve considering moderate-to-severe acute pancreatitis as positive and mild acute pancreatitis as negative, we found that NLR was more sensitive in identifying moderate-to-severe cases compared to MCTSI (Figure 1).

## Discussion

**Figure 2:**
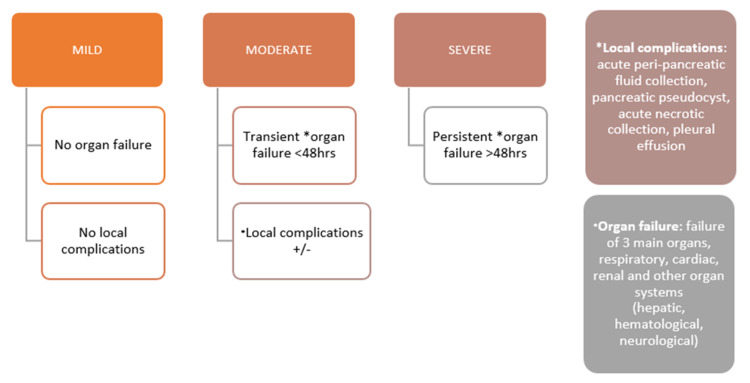
Classification of acute pancreatitis according to the 2012 revised Atlanta criteria.

In this study, we have attempted to highlight the effectiveness of NLR in assessing the severity of acute pancreatitis. Multiple studies have compared other scoring systems for the diagnosis and prognosis of acute pancreatitis utilizing clinical and radiological scores [10-12]. The presenting symptoms of pancreatitis include sudden-onset severe abdominal pain, nausea, and vomiting, with the most common causes being gallstones and alcohol abuse [13-15]. In this study, we included all acute pancreatitis cases irrespective of the cause and compared MCTSI and NLR considering the revised Atlanta classification as the gold standard. In this study, NLR was calculated using complete blood count tested on admission, and MCTSI (Table 2) was calculated for all patients once a contrast-enhanced CT scan was performed. Subsequently, the patient was classified according to the revised Atlanta classification given the laboratory workup and clinical course of the patient during the hospital stay (Figure 2).

**Table 2:** Modified CT severity index score. CT: computed tomography

Modified CT severity index score	
Prognostic indicator	Points
Pancreatic inflammation	
Normal pancreas	0
Intrinsic pancreatic abnormalities with/without inflammatory changes in peripancreatic fat	2
Pancreatic or peripancreatic fluid collection or peripancreatic fat necrosis	4
Pancreatic necrosis	
None	0
<30%	2
≥30%	4
Extrapancreatic complications	
One or more pleural effusion, ascites, vascular complications, parenchymal complications, or gastrointestinal tract involvement	2
As per MCTSI	Mild pancreatitis – modified CTSI 0-2 points Moderate pancreatitis – modified CTSI 4-6 Severe pancreatitis – modified CTSI 8-10

Among radiological scoring systems, two widely used scores, namely, CT severity index (CTSI) and MCTSI, are frequently utilized for the assessment of the severity of acute pancreatitis. Of these, MCTSI is more sensitive in categorizing moderate-to-severe disease [10,12]. Previous studies have compared scoring systems such as the modified Marshall score, the BISAP score, the APACHE II score, and the SOFA score considering radiological scores such as the Balthazar score, CTSI, and MCTSI as the gold standard for assessing the severity of the disease or vice versa [16-18]. Among clinical scoring systems, the BISAP and NLR were found to be preferable as an early determinant of disease severity [19]. According to Zahorec et al., NLR is an important marker of the degree of affliction and is an easily measurable parameter that can be widely used as a prognostic marker in numerous diseases [20]. Li et al. reported a correlation between the severity of pancreatitis and the prognostic value of inflammatory markers including NLR [21]. In addition, according to a meta-analysis published in 2020, NLR is a comprehensive biomarker with easy and rapid availability of results that may help predict the extent of inflammatory progression in acute pancreatitis and various other diseases [22]. NLR is elevated in acute pancreatitis according to the disease severity and classifies patients accordingly. As per the published data regarding the statistical value of NLR, the sensitivity and specificity are 79% (73-84%) and 71% (59-80%), respectively. On the contrary, in our study, the specificity was unusually low (13.6%). Therefore, NLR is a good predictor for assessing the severity of mild and severe cases and MCTSI is a good predictor for severe cases.

Given the above discussion, we find that in all clinical scoring systems and radiological criteria for diagnosis and prognostication of acute pancreatitis, there is a considerable burden on both patients and physicians. These lead to delay in the prediction and allocation of the level of care given the disease progression and outcome. Using NLR we can easily predict these in a short period and with limited resources, for example, in resource-limited or resource-poor rural areas (nonurban settings).

## Conclusions

NLR has good concordance with the revised Atlanta classification and assesses the disease severity, especially in moderate-to-severe cases of acute pancreatitis compared to MCTSI. Based on our results, we can conclude that NLR being readily available can be used in acute and/or resource-poor settings to predict the severity of acute pancreatitis, making it an effective marker that can help in early and timely diagnosis and treatment initiation, thereby reducing morbidity and mortality.
